# Alexidine as a Potent Antifungal Agent Against *Candida HemeuloniiSensu Stricto*

**DOI:** 10.1021/acsomega.4c11382

**Published:** 2025-03-20

**Authors:** Larissa Rodrigues Pimentel, Fabiola Lucini, Gabrieli Argueiro da Silva, Simone Simionatto, Luana Rossato

**Affiliations:** Health Sciences Research Laboratory, Federal University of Grande Dourados, Dourados, Mato Grosso do Sul 79825-070, Brazil

## Abstract

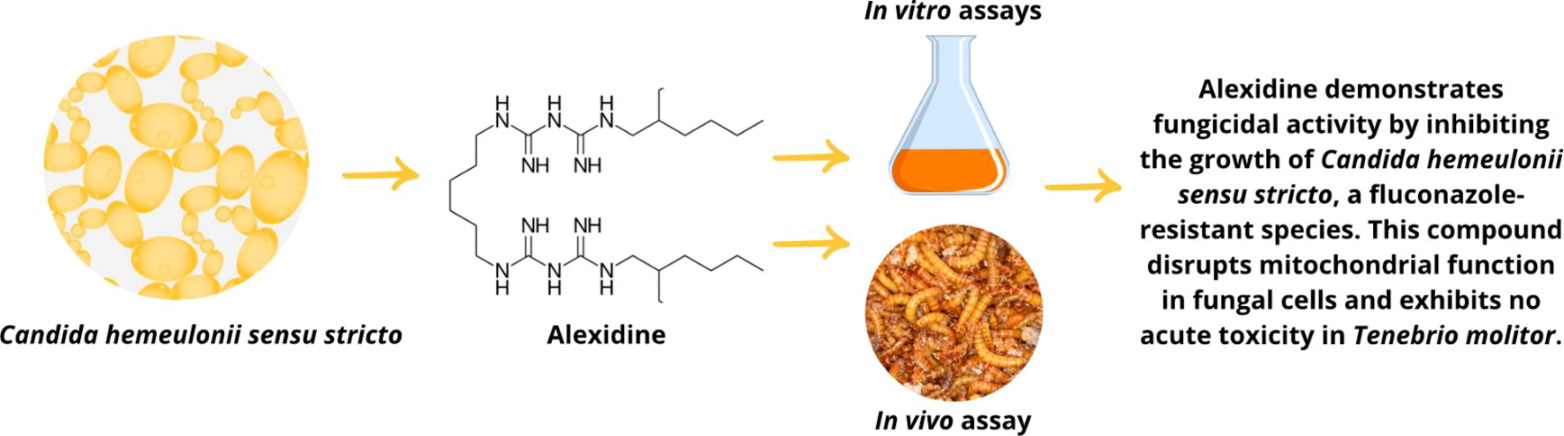

The increasing prevalence of infections by*Candida hemeuloniisensu stricto*, particularly due to its resistance to standard antifungal therapies, represents a significant healthcare challenge. Traditional treatments often fail, emphasizing the need to explore alternative therapeutic strategies. Drug repurposing, which reevaluates existing drugs for new applications, offers a promising path. This study examines the potential of repurposing alexidine dihydrochloride as an antifungal agent against*C. hemeuloniisensu stricto*. Minimum Inhibitory Concentration (MIC) and Minimum Fungicidal Concentration (MFC) values were established using broth microdilution methods. To further assess antifungal activity, different assays were conducted, including growth inhibition, biofilm inhibition, biofilm eradication, and cell damage. Checkerboard assays were employed to study the compound’s fungicidal potential and interactions with other antifungals. Additional tests, sorbitol protection assay, efflux pump inhibition, cell membrane permeability assays, and nucleotide leakage were performed. *In vivo* efficacy and safety were evaluated in*Tenebrio molitor* larvae. Alexidine demonstrated fungicidal activity against*C. hemeuloniisensu stricto*, with an MIC of 0.5 μg/mL. Biofilm formation was significantly inhibited, with a reduction of 78.69%. Mechanistic studies revealed nucleotide leakage, indicating membrane impact, but no significant protein leakage was detected. *In vivo*, alexidine displayed a favorable safety profile, with no evidence of hemolysis or acute toxicity in the *T. molitor* model. These findings support alexidine as a strong candidate for antifungal drug repurposing, especially for treating*C. hemeuloniisensu stricto* infections. Its efficacy in inhibiting growth and biofilm formation, combined with a positive safety profile, underscores its potential for clinical development as an antifungal therapy.

## Introduction

The rise of infections caused by species in the*Candida hemeulonii*complex, including*C. hemeuloniisensu stricto*,*C. hemeuloniivar. vulnera*, and*C. duobushemeulonii*, presents a growing challenge in healthcare settings, particularly among immunocompromised patients.^1,2^ These yeasts have been increasingly identified in tropical regions,^3,4^ and exhibit concerning traits, such as multidrug resistance,^5,6^ and a strong ability to adhere to prosthetic materials.^6,7^

Although*C. hemeulonii* complex infections have been rarely reported since their discovery, their clinical relevance has grown due to their intrinsic resistance to fluconazole and reduced susceptibility to amphotericin B, posing significant challenges in antifungal therapy.^8^*C. hemeulonii* complex species often demonstrate high Minimum Inhibitory Concentrations (MICs) for fluconazole, a primary antifungal agent used in treating candidemia and other *Candida* infections,^9,10^ as well as reduced susceptibility to amphotericin B, commonly used in severe or refractory cases. Although echinocandins remain effective for most isolates, cases of resistance have also been documented.^11^

Consequently, there is an urgent need to explore alternative therapeutic strategies to effectively combat these infections. Drug repurposing, which entails reevaluating existing pharmaceuticals for novel therapeutic uses, has emerged as a promising strategy to address this escalating challenge.^12^ This approach can expedite the identification of effective treatments by leveraging known safety profiles and pharmacodynamics, offering a faster path to clinical application.^13,14^

Alexidine dihydrochloride, a bis-biguanide compound, is a well-established antibacterial agent also recognized for its anti-inflammatory and anticancer properties. It induces apoptosis by inhibiting the mitochondrial tyrosine phosphatase PTPM1.^15^ Currently, alexidine is used in mouthwash as an antiplaque agent and is applied in endodontic treatments to effectively remove biofilms.^16^ Recent studies have shown that alexidine exhibits antifungal activity against some species, including*C. albicans*,*Trichophyton mentagrophytes*,*C. auris*, and*Aspergillus fumigatus*. Notably, *Trichophyton* spp. have displayed acquired resistance to terbinafine and itraconazole, which are traditionally the drugs of choice for treating dermatophyte infections.*C. auris*, a multidrug-resistant pathogen, is among the most invasive human pathogens, alongside*C. albicans* and*A. fumigatus* both of which also show significant drug resistance.^16−18^ This suggests that alexidine could be a promising candidate for treating candidiasis, as it may inhibit yeast adhesion, biofilm formation, and other pathogenic traits crucial for managing fungal infections.^19^

This study aims to evaluate the potential of alexidine as a repositioned antifungal agent for developing effective treatments against*C. hemeuloniisensu stricto* infections.

## Results

### Minimum Inhibitory Concentration (MIC)

The MIC of alexidine against*C. hemeuloniisensu stricto* was found to be 0.5 μg/mL. The MIC values for amphotericin B, fluconazole, and micafungin were determined as 4 μg/mL, 16 μg/mL, and 0.25 μg/mL, respectively. At its MIC of 0.5 μg/mL, alexidine displayed fungicidal activity against*C. hemeuloniisensu stricto*. However, when alexidine was combined with amphotericin B, fluconazole, or micafungin, no synergistic effects were observed against the*C. hemeuloniisensu stricto* isolate (Table 1).

**Table 1:** Minimum Inhibitory Concentration of Alexidine and Antifungal Agents against*C. haemuloniisensu stricto*, alone and in Combination

	MIC^a^ (μg/mL)					
	Alexidine		Test Agent			
Test agent	Alone	Combined	Alone	Combined	ΣFICI^b^	Interaction
Amphotericin B	0.5	1	4	0.5	2.12	IND
Fluconazole	0.5	0.5	16	2	1.12	IND
Micafungin	0.5	0.5	0.25	0.25	2.00	IND

a MIC: Minimum Inhibitory Concentration.

b ΣFICI (fractional inhibitory concentration index) is used to measure the interaction between the tested combinations. ΣFICI interpretation corresponded to the following definitions: synergism (SYN), ΣFICI ≤ 0.5; additivity (ADD), ΣFICI > 0.5 and ≤ 1; and indifference (IND), ΣFICI > 1 and ≤ 4; antagonism (ANT), ΣFICI ≥ 4.

### Inhibition Growth Assay

**Figure 1:**
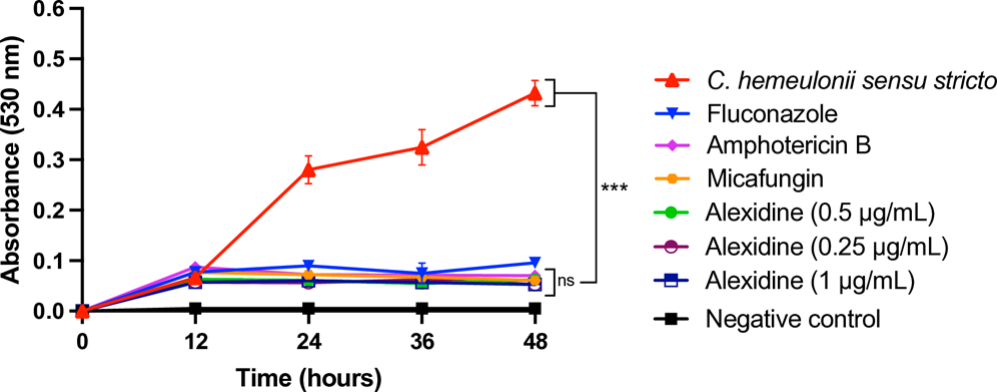
Growth curve of*C. hemeuloniisensu stricto* in the presence and absence of alexidine. The test was conducted at alexidine concentrations of 2× MIC (1.0 μg/mL), MIC (0.5 μg/mL), and 0.5× MIC (0.25 μg/mL). The negative control represents the absence of yeast. Not significant (ns). ***p < 0.001.

The efficacy of alexidine in inhibiting the growth of*C. hemeuloniisensu stricto* revealed a reduction in fungal growth after 12 h of incubation compared to the control (growth in the absence of any drug). This statistically significant difference underscores the inhibitory effect of alexidine at all tested concentrations (2× MIC, MIC, and 0.5× MIC) (Figure 1). Moreover, no significant differences were found between the alexidine concentrations, amphotericin B, and micafungin, suggesting a comparable antifungal effect among these treatments.

### Alexidine Inhibited Biofilm Formation

**Figure 2:**
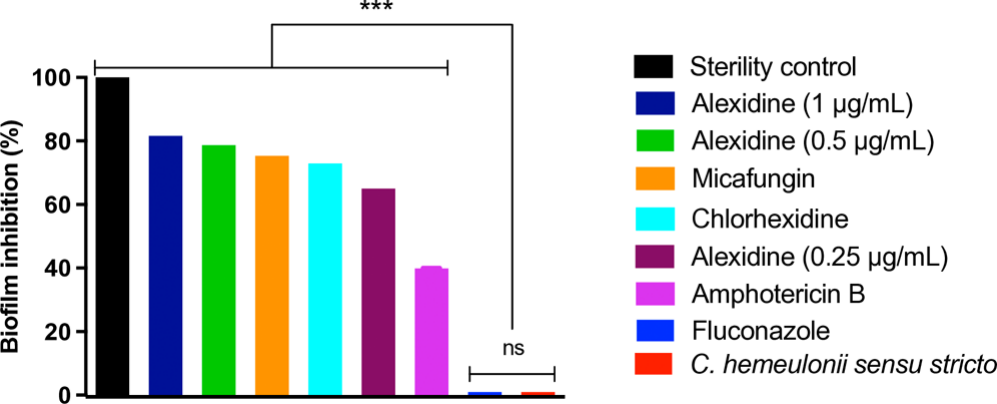
Biofilm formation inhibition test. The test was conducted at alexidine concentrations of 2× MIC (1.0 μg/mL), MIC (0.5 μg/mL), and 0.5× MIC (0.25 μg/mL). Sterility control (medium only). Not significant (ns). ***p < 0.001.

Alexidine demonstrated a notable inhibitory effect on biofilm formation, achieving an inhibition rate of 78.69% at a concentration of 0.5 μg/mL, which was higher than that of micafungin (75.27%), chlorhexidine (72.93%), and amphotericin B (39.9%). Notably, no significant differences were observed between the inhibition rates of alexidine (at concentrations of 2× MIC, MIC, and 0.5× MIC), micafungin, chlorhexidine, and amphotericin B, suggesting comparable efficacy among these treatments (Figure 2).

### Biofilm Eradication Assay

**Figure 3:**
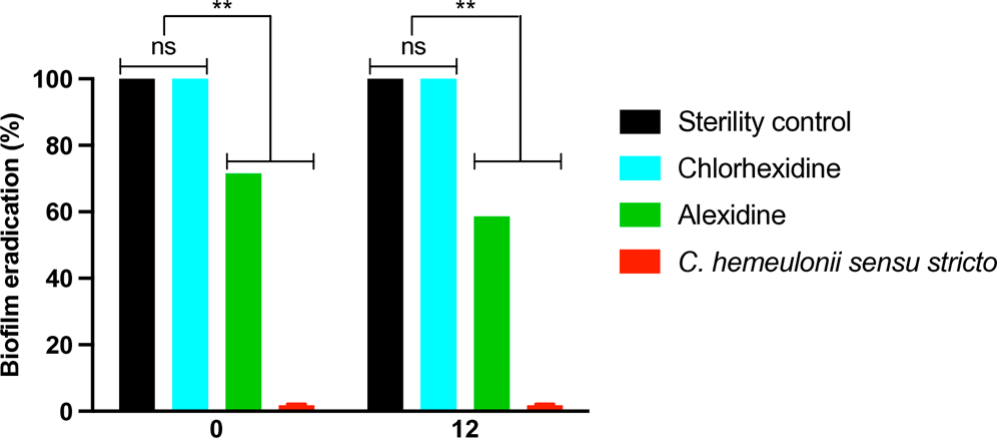
Biofilm eradication. The biofilm eradication test was conducted using alexidine (0.5 μg/mL), chlorhexidine (0.12%), sterility control (only peptone water), and*C. hemeuloniisensu stricto*. Not significant (ns). **p < 0.01.

The biofilm eradication assay was performed on*C. hemeuloniisensu stricto* using alexidine at a concentration of 0.5 μg/mL, with chlorhexidine (0.12%) included as a comparative disinfectant. Alexidine achieved a biofilm eradication rate of 71.42% at 15 min and 58.53% at 45 min, whereas chlorhexidine showed eradication rates of 100.00% at 15 and 45 min (Figure 3).

### Cell Damage

**Figure 4:**
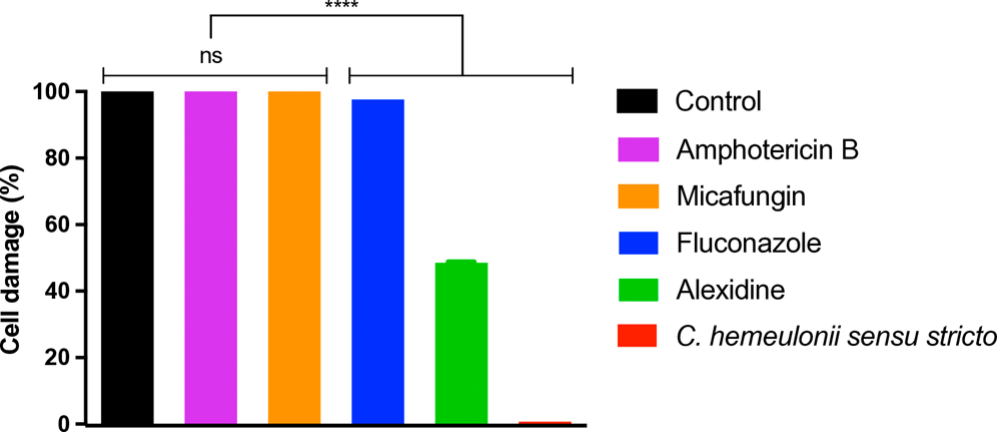
Damage to mitochondria of*C. hemeulonii* sensu stricto cells in the presence and absence of alexidine. The test was performed at MIC concentration (0.5 μg/mL). The control represents the absence of yeast. Not significant (ns). ****p < 0.0001.

The results indicate that while amphotericin B and micafungin demonstrated superior efficacy compared to alexidine, the use of alexidine still resulted in a significant impact on mitochondrial functionality. Specifically, alexidine induced mitochondrial dysfunction in 48.73% of*C. hemeuloniisensu stricto* cells, leading to notable disruption in cellular respiration. These findings emphasize the potential of alexidine as a valuable option in targeting mitochondrial integrity, even when its effectiveness is slightly lower than that of amphotericin B and micafungin (Figure 4).

### Sorbitol Protection Assay

The impact of alexidine on the cell wall integrity of*C. hemeuloniisensu stricto* showed that alexidine did not compromise the cell wall structure via the sorbitol-dependent pathway, as no increase in MIC was observed when sorbitol was present. According to established criteria, cell wall damage is indicated when the MIC of a compound rises in the presence of sorbitol compared to its absence. These findings are detailed in Table 2, confirming that alexidine’s antifungal action does not involve direct disruption of cell wall integrity.

**Table 2:** Minimum Inhibitory Concentration of Alexidine against*C. haemuloniisensu stricto* in the Presence and Absence of Sorbitol and Promethazine

	Alexidine				Micafungin			
Strain	MIC^a^ (48h)		MIC (72h)		MIC (48h)		MIC (72h)	
*C. hemeuloniisensu stricto*	**S-**	**S+**	**S-**	**S+**	**S-**	**S+**	**S-**	**S+**
	0.5	0.5	0.5	0.5	1	≥8	1	≥8
	**P-**	**P+**	**P-**	**P+**	-	-	-	-
	0.5	64	0.5	64	-	-	-	-

a MIC: minimum inhibitory concentration; S-: absence of sorbitol; S+: presence of sorbitol; P−: absence of promethazine; P+: presence of promethazine.

### Efflux Pump Inhibition Assay

The study also included tests to assess whether alexidine could inhibit efflux pumps, a common mechanism contributing to resistance. For the efflux pump assay,*C. hemeuloniisensu stricto* cells were exposed to alexidine at a concentration of 0.5 μg/mL, both with and without the addition of promethazine (128 μg/mL), an established efflux pump inhibitor. Results showed that the antifungal activity of alexidine remained consistent at both 48 and 72 h, suggesting that efflux pumps were not activated under these conditions. Specifically, the presence of promethazine did not influence the activity of alexidine, indicating no involvement of efflux pumps in mediating resistance (Table 2).

### Alteration of Cell Membrane Permeability

To evaluate the integrity of the fungal cell membrane, protein leakage was measured as an indicator of potential damage or disruption caused by alexidine treatment. Protein leakage is a commonly used marker to assess whether compounds compromise the cellular structure, as it reflects the ability of the membrane to retain essential macromolecules. In this study, cells treated with alexidine showed no detectable protein leakage, indicating that the membrane remained intact and functional. This suggests that, under the tested conditions, alexidine did not cause significant structural damage to the fungal cell membrane, preserving its ability to maintain cellular homeostasis.

### Nucleotide Leakage

**Figure 5:**
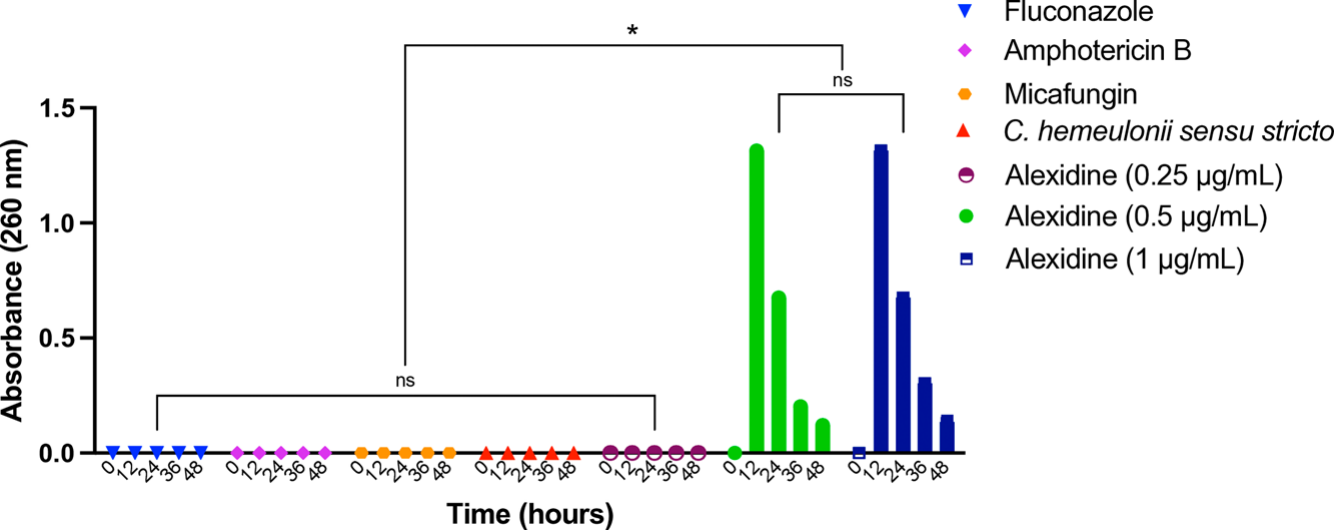
Nucleotides extravasated from*C. hemeuloniisensu stricto* after treatment with alexidine. The test was conducted at alexidine concentrations of 2× MIC (1.0 μg/mL), MIC (0.5 μg/mL), and 0.5× MIC (0.25 μg/mL). Not significant (ns). *p < 0.05.

To further investigate cell membrane integrity and assess the potential cytotoxic effects of alexidine, nucleotide leakage was measured. Results indicated that cultures treated with alexidine exhibited nucleotide leakage, with peak levels observed at 12 h. The statistical analysis revealed significant differences between the groups treated with alexidine at 0.5 and 1 μg/mL and the control group (*C. hemeuloniisensu stricto* only), highlighting its impact on membrane integrity (Figure 5). Additionally, significant differences were also observed between the alexidine-treated groups (0.5 and 1 μg/mL) and the other antifungals tested (Figure 5).

### Hemolysis Assay

**Figure 6:**
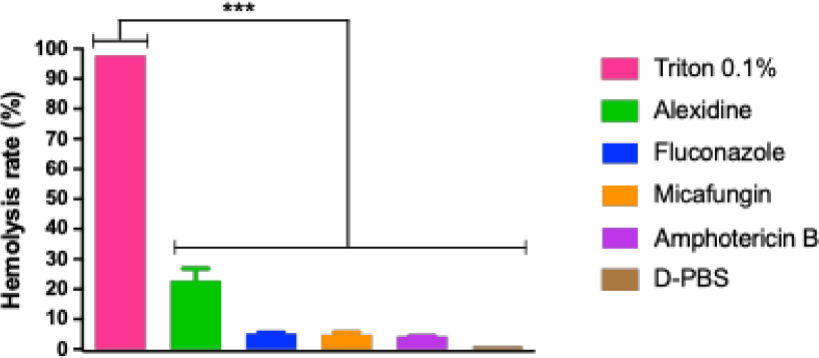
Hemolysis assay. The relative hemolysis rate in commercially sourced defibrinated sheep blood after incubation with alexidine (MIC 0.5 μg/mL), D-PBS (negative control), and 0.1% Triton (positive control). ***p < 0.001.

Hemolysis assays were conducted to evaluate the hemocompatibility of alexidine. At a concentration of 0.5 μg/mL, alexidine induced approximately 20% hemolysis, which was significantly lower than the positive control (Triton). Although a 20% hemolysis rate was observed, alexidine did not differ statistically from fluconazole, micafungin, or amphotericin B. These results suggest that alexidine exhibits good hemocompatibility with minimal hemolytic activity (Figure 6).

### *In Vivo* Survival Assay and Antifungal Treatment

**Figure 7:**
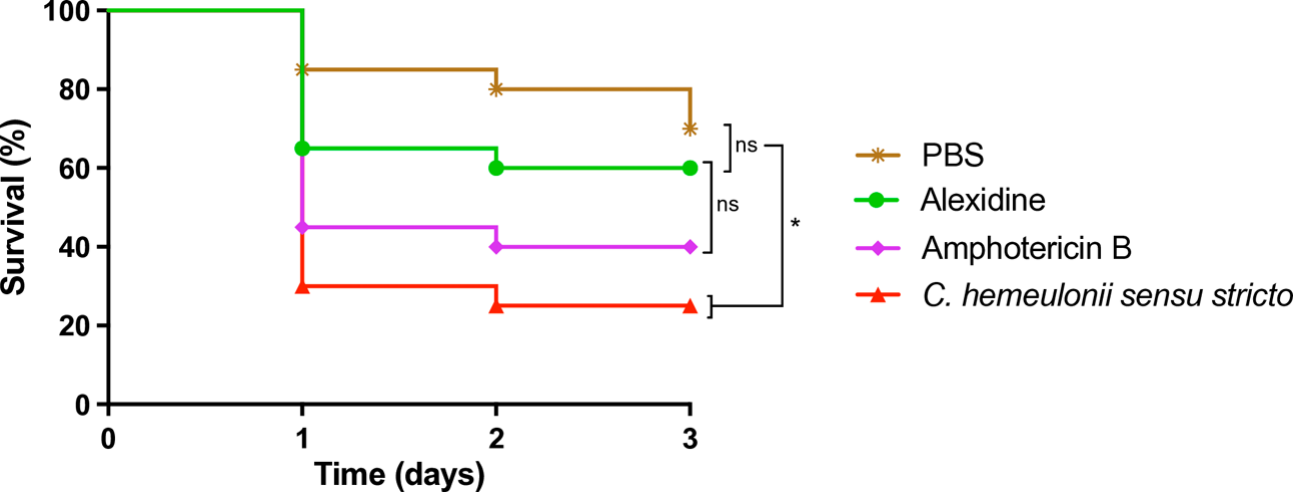
Survival curves of *T. molitor* larvae infected with*C. hemeuloniisensu stricto*. Groups of 25 larvae were infected with 5 μL of a fungal suspension at a concentration of 2.5 × 10^5^ cells/mL. Simultaneously, 5 μL of the assigned treatment was administered: alexidine at MIC (0.5 μg/mL) and amphotericin B (4 μg/mL). The assay included a negative control group, in which larvae were injected with PBS, as well as a growth control group, where larvae were infected with*C. hemeuloniisensu stricto* but received no treatment. *p < 0.05, determined by the log-rank test.

Survival curves were generated and analyzed to assess differences in survival rates among the treatment groups over time. The PBS control group displayed a steady decrease in survival over the 3-day observation period. In contrast, the amphotericin B-treated group showed a more pronounced decline, with survival rates dropping to approximately 40% by day 3. The group treated with alexidine exhibited a slower decline in survival, maintaining approximately 61% at the end of the 3-day period (Figure 7).

## Discussion

The severity of *Candida* infections is escalating due to rising antimicrobial resistance and a scarcity of effective treatment options. Drug repurposing offers a viable approach to address these challenges, offering the potential to expedite the discovery of new therapeutic uses for existing drugs. This study explores the antifungal potential of alexidine against the*C. hemeuloniisensu stricto* strain. Specifically, the investigation assessed the impact of alexidine on various growth and virulence-related factors, including biofilm formation, membrane integrity, and mitochondrial function.

In this study, alexidine exhibited fungicidal activity with an MIC of 0.5 μg/mL against*C. hemeuloniisensu stricto*, which is comparable to micafungin (0.25 μg/mL) and significantly lower than fluconazole (16 μg/mL). Fluconazole resistance in*C. hemeulonii* is well-documented, often leading to treatment failure in clinical settings.^1^ Amphotericin B, despite its broad-spectrum activity, shows variable efficacy against*C. hemeulonii*, requiring higher MICs in some strains.^10^ Our findings indicate that alexidine matches or surpasses the activity of conventional antifungals, reinforcing its potential for drug repurposing. Mamouei et al.^16^ conducted a screening of the New Prestwick Chemical Library, revealing that alexidine suppresses growth by at least 50% in several*Candida* species (including*C. albicans* and*C. auris*), as well as *Cryptococcus neoformans* and*A. fumigatus*. Furthermore, alexidine’s efficacy against various filamentous fungi, including*Fusarium solani* and*F. oxysporum* was documented.^20^ Together with previous studies, our results further underscore the broad-spectrum antifungal potential of alexidine, extending its efficacy to*C. hemeuloniisensu stricto*.

Biofilms exhibit unique developmental characteristics, making them significantly more challenging to treat than planktonic cells.^21^ This complexity is especially relevant given the widespread issue of antimicrobial resistance, which emphasizes the critical need to evaluate both existing and novel antifungal agents for efficacy against biofilm-associated cells.^22^ In this context, our study’s findings on alexidine are particularly compelling, as it demonstrated a significant reduction in biofilm formation. Building on previous research that highlighted the antibiofilm potential of alexidine in species such as*A. fumigatus*,*C. neoformans*, and multiple *Candida* species. Additionally, alexidine successfully eradicated preformed biofilms.^16,23^ These findings suggest that alexidine could be a valuable option for treating biofilm-associated infections, particularly in drug-resistant species.

Unlike amphotericin B, which primarily targets ergosterol, alexidine’s antifungal mechanism appears to involve mitochondrial dysfunction and nucleotide leakage without direct membrane destabilization. This aligns with previous studies indicating that alexidine inhibits mitochondrial phosphatase PTPMT1, leading to membrane depolarization and apoptosis.^15,24,25^ Notably, its activity does not seem to be affected by drug efflux pumps, a common mechanism of resistance in azoles.^26^ This suggests that alexidine may be effective against multidrug-resistant fungal strains. In this context, our findings suggest that alexidine’s mechanism of action may extend beyond biofilm inhibition, potentially affecting key cellular components essential for biofilm maintenance. Notably, our results indicate that alexidine induces mitochondrial dysfunction in*C. hemeuloniisensu stricto*, which could contribute to biofilm disruption by impairing energy production and cellular homeostasis.

The cytotoxicity of alexidine has been previously documented in the literature.^16,23,27^ While alexidine exhibited approximately 20% hemolysis, this level was not statistically different from amphotericin B, micafungin, or fluconazole. However, prior research has reported cytotoxic effects in mammalian cells at concentrations above 14.7 μg/mL.^16,18^ This highlights the need for further toxicity evaluations, particularly in mammalian models, to determine the therapeutic window and safety profile of alexidine for potential clinical applications.

The survival rate of*T. molitor* larvae treated with alexidine compared to amphotericin B suggests potential *in vivo* efficacy. Previous studies using mammalian models have demonstrated that alexidine achieves both clinical and mycological clearance of fungal infections caused by*Trichophyton mentagrophytes*.^18^ By day 7 post-treatment, lesions were completely healed, showing a substantial reduction in infection following topical administration. Although*T. molitor* models provide valuable preliminary toxicity insights, further murine model studies are required to confirm the clinical relevance of these findings.

There is an urgent need to identify and characterize new agents with efficacy against *C. hemeulonii sensu stricto*. Our findings highlight alexidine as a promising candidate for addressing *C. hemeulonii sensu stricto* infections. Its existing clinical approval offers a valuable advantage, potentially accelerating the repurposing process and enabling more rapid integration into antifungal treatment strategies.

Additionally, alexidine disrupted mitochondrial function, as indicated by nucleotide leakage, yet did not cause significant protein leakage, suggesting that its antifungal mechanism does not involve direct cell membrane destabilization. Furthermore, in *in vivo* assays using the *T. molitor* model, alexidine showed no acute toxicity. However, its efficacy was comparable to amphotericin B, indicating that further studies are necessary to optimize its therapeutic potential and explore its mechanistic interactions with fungal cells. Although alexidine’s broad antimicrobial activity makes it a promising candidate for drug repurposing, additional investigations are required to confirm its clinical applicability, particularly regarding pharmacokinetics, cytotoxicity in mammalian cells, and potential resistance mechanisms. This study highlights alexidine as a viable antifungal agent, paving the way for future research to explore its full potential in antifungal therapy.

## Methods

### Strain and Culture Conditions

The*C. hemeuloniisensu stricto* (132/23) strain utilized in this study was obtained from the strain library of the Center for Studies in Applied Medical Mycology (CSAMM) at the Health Sciences Research Laboratory (HSRL), Universidade Federal de Grande Dourados (UFGD). This strain had been previously characterized for its biofilm production and identified through sequencing of the Internal Transcribed Spacer (ITS) region of rDNA.^1,28^ To assess the interaction between tafenoquine and antifungal agents, we selected this strain, an isolate obtained from a blood culture, which demonstrated resistance to amphotericin B.^9^ The strain was stored in 20% glycerol at −80 °C until required for experimentation. For the experimental procedures, the strain was cultured on Sabouraud Dextrose Agar (SDA), and incubated at 37 °C for 48 h.

### Minimum Inhibitory Concentration (MIC)

The Minimum Inhibitory Concentration (MIC) of the drug was determined using the broth microdilution method, following the guidelines set by the European Committee on Antimicrobial Susceptibility Testing (EUCAST 7.4).^29^ The sensitivity of alexidine dihydrochloride was evaluated across a concentration range of 0.25 to 128 μg/mL. Fungal suspensions were prepared, diluted in sterile distilled water, and inoculated into 96-well plates at a final density of 2.5 × 10^5^ cells/mL. The plates were incubated at 37 °C for 48 h. Antifungal activity was assessed spectrophotometrically at 530 nm, with the MIC defined as the lowest concentration of the compound that inhibited 90% of fungal growth. To determine the Minimum Fungicidal Concentration (MFC), 10 μL samples from each MIC well were transferred to Sabouraud agar plates and incubated at 37 °C for 48 h. The MFC was defined as the lowest concentration at which no visible fungal growth occurred. Amphotericin B, at concentrations ranging from 0.03 to 16 μg/mL, served as a resistance control.

### Checkboard Assay

The checkerboard microdilution method was employed to assess the interaction of alexidine with fluconazole, amphotericin B, and micafungin.^30^ The concentration ranges tested were 0.25 to 128 μg/mL for alexidine dihydrochloride, 0.125 to 64 μg/mL for fluconazole, 0.031 to 16 μg/mL for amphotericin B, and 0.015 to 8 μg/mL for micafungin. For each plate setup, 50 μL of alexidine dihydrochloride was added horizontally, and 50 μL of the antifungal agent was added vertically in a 96-well flat-bottom plate. Then, 100 μL of inoculum (final concentration of 10^5^ cells/mL) was added to each well containing the compounds. Positive controls were included in eight wells containing only 2× RPMI medium supplemented with 2% glucose and 165 mM MOPS (pH 7.0), while sterility controls were performed in eight wells containing only the medium. The plates were incubated for 48 h at 37 °C.

The interaction between the compounds was quantified using the Fractional Inhibitory Concentration Index (FICI), calculated as follows: FICI = [MIC of alexidine dihydrochloride in combination]/[MIC of alexidine dihydrochloride alone] + [MIC of drug in combination]/[MIC of drug alone]. Each plate’s FICI was calculated for all interaction concentrations and classified according to the criteria described in ref (31) synergy (FICI ≤ 0.5), indifference (0.5 < FICI ≤ 4), or antagonism (FICI > 4).

### Inhibition Growth Assay

For the growth curve analysis, an inoculum of 2.5 × 10^5^ cells/mL was prepared. This suspension was combined with alexidine dihydrochloride (MIC 0.5 μg/mL) and 2× RPMI medium supplemented with 2% glucose and 165 mM MOPS (pH 7.0), which served as the growth control. The negative control contained only the medium without yeast, and amphotericin B was included as the resistance control. The plates were incubated at 37 °C, and absorbance was measured at 530 nm at 0, 12, 24, 36, and 48 h. Turbidity was plotted against incubation time, and growth rate curves were analyzed to evaluate the fungicidal effects of alexidine dihydrochloride.^32^ All experiments were performed in triplicate.

### Antibiofilm Activity

Biofilm inhibition was evaluated following a previously established method,^33^ with modifications. In brief, a standardized inoculum of*C. hemeuloniisensu stricto* (2.5 × 10^5^ cells/mL) in 2× RPMI medium, enriched with 2% glucose and buffered with 165 mM MOPS at pH 7.0, was combined with varying concentrations of the selected compound (alexidine dihydrochloride: 0.25–128 μg/mL). This suspension was added to the wells of 96-well polystyrene microtiter plates, which were then incubated without agitation at 37 °C for 48 h to promote fungal growth and biofilm formation.

After incubation, planktonic cells were carefully removed, and the biomass adhered to the plate was washed three times with distilled water. The remaining biofilm was stained with 0.1% crystal violet for 20 min. Excess dye was discarded, and the stained biomass was resuspended in 70% ethanol. Fungal growth was quantified by measuring absorbance at 595 nm using a microplate reader (iMarkTM Microplate, Bio-Rad, São Paulo, SP, Brazil). The controls consisted of*C. hemeuloniisensu stricto* cells in 2× RPMI medium containing 2% glucose and 165 mM MOPS (pH 7.0), as well as the medium without yeast.

The percentage of biofilm inhibition was calculated relative to the untreated biofilm (considered 100% biofilm formation) and the sterility control containing only medium (considered 0% biofilm). Biofilm inhibition was determined according to the formula provided:^34^
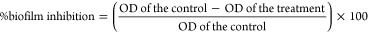


### Biofilm Eradication Assay

*Candida hemeuloniisensu stricto* cells were cultured on SDA for 48 h and then resuspended in peptone water (HiMedia) at a concentration of 10^5^ CFU/mL. Hospital devices, specifically endotracheal tubes, were immersed in this yeast suspension and incubated for 48 h at 37 °C to promote biofilm formation. A parallel control group was prepared using endotracheal tubes that were exposed solely to peptone water without yeast, serving as the sterility control. After the incubation period, the endotracheal tubes were rinsed three times with sterile distilled water to remove nonadherent cells. The endotracheal tubes with adhered biofilm were then treated with 4 μg/mL of tafenoquine, 0.12% chlorhexidine, and peptone water (untreated control) for 15 and 45 min.

Biofilm was collected from the endotracheal tubes using physical agitation. After plating the samples on SDA, they were incubated at 37 °C, and the CFU count was determined. The biofilm eradication percentage was calculated with the formula % biofilm eradication = [(CFU of untreated biofilm – CFU of treated biofilm)/CFU of untreated biofilm] × 100, comparing CFU counts of treated samples to the untreated control.^22^

### Cell Damage

To assess and quantify cellular damage caused by alexidine dihydrochloride, we performed an MTT assay (3-(4,5-dimethylthiazol-2-yl)-2,5-diphenyltetrazolium bromide) following established protocols.^35,36^ After incubating for 24 h, the plates were centrifuged at 40 g for 10 min at room temperature, and the supernatant was carefully removed. The resulting cell pellets were then exposed to 200 μL of an aqueous MTT solution (0.05 mg/mL) and incubated for an additional 3 h at 37 °C. Postincubation, the plates were centrifuged again, and the formazan crystals were dissolved using 150 μL of isopropyl alcohol. For absorbance measurements, 100 μL from each well was transferred to fresh wells, where absorbance was read at both 595 nm (A595) and 655 nm (A655). The extent of cellular damage was calculated and presented graphically in a bar chart. Each experiment was conducted in triplicate. The formula used to determine cellular damage is as follows:
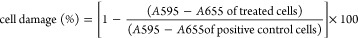


### Sorbitol Protection Assay

Following a previously established methodology, we assessed the osmoprotective effect of sorbitol.^37,38^ A serial microdilution was performed in a sterile 96-well microplate containing 2× RPMI medium with 2% glucose and 165 mM MOPS (pH 7.0), supplemented with 0.8 M sorbitol. The alexidine dihydrochloride stock solution was prepared at concentrations ranging from 0.25 to 128 μg/mL, with micafungin included as a positive control. MIC values were assessed after incubation at 37 °C for 48 and 72 h. Each test was conducted in duplicate.

### Efflux Pump Inhibition Assay

To assess whether alexidine dihydrochloride can inhibit these efflux pumps, we conducted a phenotypic susceptibility assay utilizing promethazine, a known inhibitor of plasma membrane efflux pumps.^26^ This assay was performed with alexidine dihydrochloride at concentrations ranging from 0.25 to 128 μg/mL, while incorporating subinhibitory concentrations of promethazine (128 μg/mL) into the fungal inoculum to observe any potential synergistic effect on efflux pump inhibition.

### Alteration of Cell Membrane Permeability

Cell membrane permeability was evaluated using the BCA Protein Assay Kit.*C. hemeuloniisensu stricto* cells were suspended in sterile distilled water at a concentration of 2.5 × 10^5^ cells/mL. The inoculum was then combined with alexidine dihydrochloride (MIC: 0.5 μg/mL) and incubated at 37 °C for 0, 12, 24, 36, and 48 h. Following each incubation period, samples were centrifuged at 908 g for 5 min at 4 °C. After centrifugation, 25 μL of the supernatant was transferred to a flat-bottom 96-well plate, where 200 μL of the BCA working reagent was added to each well. The plate was shaken for 30 s and incubated at 37 °C for 30 min. Absorbance was then measured at 595 nm.

To account for background absorbance, the average absorbance of the control wells was subtracted from that of the treatment wells. For each treatment, a blank containing only the treatment and the BCA working reagent was also prepared, and this blank value was subtracted from the treatment results. The protein concentration (μg/mL) was determined using a linear formula derived from the kit’s calibration curve, allowing for a direct correlation between absorbance and the amount of protein released by the yeast cells.^39^

### Nucleotide Leakage

The methodology adhered to a previously established procedure.^40^*C. hemeuloniisensu stricto* cultures were grown on SDA at 37 °C for 48 h. After incubation, cells were suspended in 0.9% saline to reach a concentration of 2.5 × 10^5^ cells/mL. The microorganism was then exposed to alexidine dihydrochloride at its MIC (0.5 μg/mL) for intervals of 0, 12, 24, 36, and 48 h. Cells incubated solely with 0.9% saline served as a negative control, while amphotericin B was included as a resistance control. The supernatants from these suspensions were centrifuged at 1300*g* for 15 min, and absorbance was measured at 260 nm to assess cellular response. Each procedure was conducted in triplicate to ensure reproducibility.

### Hemolysis Assay

Hemolysis was evaluated to determine the hemocompatibility of alexidine dihydrochloride, focusing on its potential application as a antifungal agent. Following a previously described method,^41^ commercially sourced defibrinated sheep blood was diluted 1:25 in sterile PBS, and 250 μL of this diluted blood was incubated with alexidine dihydrochloride at its MIC (0.5 μg/mL). PBS was used as the negative control, ensuring baseline compatibility, while 0.1% (v/v) Triton served as the positive control to induce complete hemolysis. The samples were incubated at 37 °C for 1 h, then centrifuged at 700*g* for 5 min to separate plasma from intact red blood cells. After centrifugation, 100 μL of the supernatant from each sample was transferred to a 96-well flat-bottom plate, and absorbance was measured at 490 nm using a microplate reader. The hemolysis ratio (%) was calculated using the following formula:
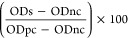


ODs: OD490 values for samples, ODnc: OD490 values for negative controls, ODpc: OD490 values for positive controls.

### *In Vivo* Survival Assay and Antifungal Treatment in the *T. Molitor* Model

To assess alexidine dihydrochloride’s efficacy in treating*C. hemeuloniisensu stricto* infection, we applied a modified version of a previously described method.^39,42^ The experimental groups were structured as follows: group 1 received only PBS (negative control); Group 2 was treated with alexidine dihydrochloride at MIC (0.5 μg/mL); and Group 3 received amphotericin B (4 μg/mL).*C. hemeuloniisensu stricto* cells, cultured on SDA at 37 °C for 48 h, were suspended in PBS to reach a density of 2.5 × 10^5^ cells/mL. A 5-μL aliquot of this cell suspension was injected into the larval hemocoel using a Hamilton syringe (Hamilton, USA), targeting the area between the third and fourth abdominal sternites. Simultaneously, 5 μL of the assigned treatment was injected. The*Tenebrio molitor* larvae were incubated at 37 °C, and survival was monitored by counting larvae responsive to touch every 24 h for a total of 72 h.

### Statistical Analysis

The Tukey test was employed to compare the outcomes of the hemolysis assay, while Kaplan–Meier survival curves were generated for*T. molitor*, with statistical significance evaluated using the log-rank test. All statistical analyses were performed in GraphPad Prism 8 software (GraphPad Software, Inc., San Diego, CA, USA), with significance defined at*p* values <0.05.

## Data Availability

The data underlying this study are available in the manuscript.
